# In Vivo Imaging of Retinal and Choroidal Morphology and Vascular Plexuses of Vertebrates Using Swept-Source Optical Coherence Tomography

**DOI:** 10.1167/tvst.11.8.11

**Published:** 2022-08-16

**Authors:** Ratheesh K. Meleppat, Christopher R. Fortenbach, Yifan Jian, Esteban Soto Martinez, Karen Wagner, Bobeck S. Modjtahedi, Monica J. Motta, Deepa L. Ramamurthy, Ivan R. Schwab, Robert J. Zawadzki

**Affiliations:** 1 UC Davis Eyepod Imaging Laboratory, University of California Davis, Davis, CA, USA; 2 Department of Ophthalmology & Vision Science, University of California Davis, Sacramento, CA, USA; 3 Center for Neuroscience, University of California, Davis, Davis, CA, USA; 4 Department of Ophthalmology and Visual Sciences, Carver College of Medicine, University of Iowa, Iowa City, IA, USA; 5 Casey Eye Institute, Oregon Health & Science University, Portland, OR, USA; 6 Department of Medicine and Epidemiology, School of Veterinary Medicine, University of California Davis, Davis, CA, USA; 7 Department of Entomology and UC Davis Comprehensive Cancer Center, University of California Davis, Sacramento, CA, USA; 8 Department of Research and Evaluation, Southern California Permanente Medical Group, Pasadena, CA, USA; 9 Department of Clinical Science, Kaiser Permanente Bernard J. Tyson School of Medicine, Pasadena, CA, USA; 10 Department of Surgical and Radiological Sciences, School of Veterinary Medicine, University of California, Davis, CA, USA

**Keywords:** Swept-source Optical coherence tomography, OCTA, Retina, Choroid, vertebrates

## Abstract

**Purpose:**

To perform in vivo evaluation of the structural morphology and vascular plexuses of the neurosensory retina and choroid across vertebrate species using swept-source optical coherence tomography (SS-OCT) and SS-OCT angiography (SS-OCTA) imaging.

**Methods:**

A custom-built SS-OCT system with an incorporated flexible imaging arm was used to acquire the three-dimensional (3D) retinal OCT and vascular OCTA data of five different vertebrates: a mouse (C57BL/6J), a rat (Long Evans), a gray short-tailed opossum (*Monodelphis domestica*), a white sturgeon (*Acipenser transmontanus*), and a great horned owl (*Bubo virginianus*).

**Results:**

In vivo structural morphology of the retina and choroid, as well as en face OCTA images of retinal and choroidal vasculature of all species were generated. The retinal morphology and vascular plexuses were similar between rat and mouse, whereas distinct choroidal and paired superficial vessels were observed in the opossum retina. The retinal and vascular structure of the sturgeon, as well as the pecten oculi and overlying the avascular and choroidal vasculature in the owl retina are reported in vivo.

**Conclusions:**

A high-quality two-dimensional and 3D in vivo visualization of the retinal structures and en face visualization of the retina and choroidal vascular plexus of vertebrates was possible. Our studies affirm that SS-OCT and SS-OCTA are viable methods for evaluating the in vivo retinal and choroidal structure across terrestrial, aquatic, and aerial vertebrates.

**Translational Relevance:**

In vivo characterization of retinal morphology and vasculature plexus of multiple species using SS-OCT and SS-OCTA imaging can increase the pool of species available as models of human retinal diseases.

## Introduction

The visual systems across the animal kingdom are exquisitely designed to best support unique visuoecological demands.^1^ A myriad of adaptations to the visual properties of specific environments are evident in the structure and function of the ocular design across species. Functional and anatomical differences in the ocular structures often reflect distinct selective pressures exerted by the ecological niches.^2^^,^^3^ The knowledge of retinal structure and function across species and their comparative studies shed light on the adaptations of the evolving visual system among different species. Investigating the visual systems of different vertebrates also has significance in ophthalmology and neurology research because many vertebrates serve as disease models of different ophthalmological and neurological diseases found in humans. Identifying similarities between human diseases in vertebrate models facilitates the understanding of numerous human retinal diseases and aids in the development of novel drugs, drug delivery strategies, and advancement of ophthalmological diagnostic technologies. Characterization of the structural morphology of the retina and retinal vasculature in animals is therefore essential not only to further our fundamental understanding of the visual system but also to aid in our understanding of pathophysiology and treatment of disease.

Because of the stratified structure of the retina, longitudinal observation of the retina is preferred to investigate the pathogenesis or effects of therapeutic approaches in disease models. The transparent ocular media offers a unique opportunity to noninvasively assess the retina in vivo using optical imaging modalities. Progress in modern optical imaging techniques allows evaluation with high spatial and temporal resolution leading to the extraction of critical information regarding retinal morphology and its changes with near-cellular-level resolution. Fundus photography, including fluorescein angiography has been widely used for the in vivo imaging of the retina over many decades.^4^ However, the lack the depth discrimination limits its ability to assess the retina and deeper structures.

Over the past two decades, optical coherence tomography (OCT) has emerged as a powerful non-invasive tool for the longitudinal investigation of the retina since it allows the detailed visualization of retinal morphology and vasculature at near-cellular resolution.^5^^,^^6^ OCT has become a gold standard in the clinical evaluation of retinal and optic nerve diseases. It generates high-resolution images by detecting back-reflected/backscattered near-infrared light from different layers of the retina using low coherence interferometry. Fourier-domain OCT imaging has become widely used for retinal imaging over conventional time-domain OCT because of its capability for high-speed imaging with high-sensitivity.^7^ The Fourier-domain OCT systems are generally classified as spectral-domain OCT (SD-OCT) and swept-source OCT (SS-OCT) configurations. The SS-OCT imaging has several advantages over SD-OCT, including reduced sensitivity fall-off and lower fringe washout artifacts caused by the sample motion.^8^^,^^9^ Furthermore, the availability of extremely narrow-linewidth tunable light sources with higher sweeping rate, sophisticated data acquisition, and applicability of the balanced detection enable a high-speed and deeper retinal imaging at a longer near-infrared window using an SS-OCT system.^10^^–^^12^ The capability of performing high-speed imaging, increased tissue penetration at a longer wavelength, and improved signal-to-noise ratio allows for the collection of high-quality cross-sectional and volumetric (3D) retina data using a SS-OCT system.

Optical coherence tomography angiography (OCTA) is a functional extension of the OCT imaging scheme, which allows the in vivo and label-free visualization of the depth-resolved vascular maps (utilizing flow contrast) of the retina.^13^^,^^14^ OCTA detects blood flow by measuring change (decorrelation or variance) in OCT signal in consecutive cross-sectional images (B-scans) taken from the same location. OCTA can provide several vascular maps of the retina, including those found in the superficial nerve fiber layer (NFL), intermediate plexiform layers, and deep choroidal vasculature beds. Commercially available OCT devices are helpful in investigating retinal and choroidal vascular disease in humans. Retinal imaging prototypes for large animals such as non-human primates (rhesus macaques) and pigs have also been reported.^15^^,^^16^ The instrumentation designed for use in human patients is limited in its flexibility for retinal imaging of a wide range of animal models because each species has a unique set of adjustments (animal placing, pupil size, eye focal length, etc.) that are needed to obtain reproducible and high-quality OCT images. OCT imaging is further complicated in small animals since their pupil sizes are very small (often only a few mm in size). A single and flexible imaging platform that allows imaging of retinal and choroidal anatomy, and vascular plexus of a variety of small terrestrial, aquatic, and aerial vertebrates is highly desirable in preclinical research.

Considering the intense research efforts in the field of ophthalmology aimed at understanding the retinal and choroidal vascular architectures in different species, herein, we demonstrate the application of an in-house developed SS-OCT and SS-OCT angiography (SS-OCTA) imaging system with an incorporated flexible imaging arm (gimbal scanning head and holder), for noninvasive, noncontact, in vivo retinal imaging of different small vertebrates to visualize depth-resolved retinal and choroidal morphology and vasculature.

## Materials and Methods

### Animal Handling for in Vivo Imaging

**Figure 1.**
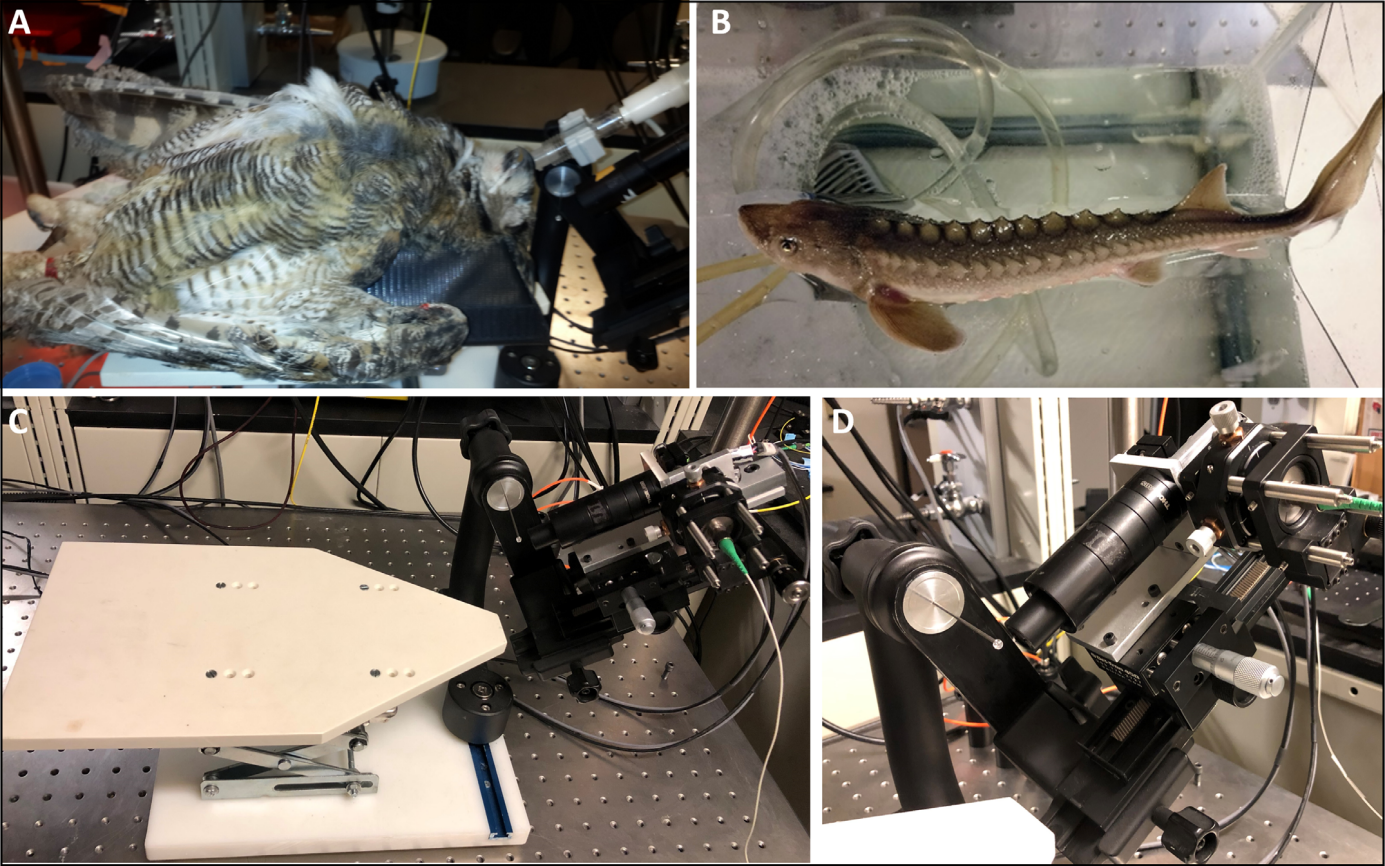
Representative photographs of (**A**) retinal imaging of anesthetized (isoflurane) great horned owl; (**B**) white sturgeon anesthetized with MS-222, with tubing used to provide oxygen throughout the imaging; (**C**) imaging platform; and (**D**) imaging head with scan and tube lens.

Vertebrates from varied phyla were imaged, including the rat (Long Evans, eight months old), mouse (C57BL/6J, six months old), gray short-tailed opossum (*Monodelphis domestica,* 11 months old), great horned owl (*Bubo virginianus,* three years old), and a white sturgeon (*Acipenser transmontanus,* 1.5 years old). All animal handling and imaging were performed in compliance with the relevant guidelines and regulations put forth by the University of California Davis Institutional Animal Care and Use Committee, the National Institute of Health, and the Association for Research in Vision and Ophthalmology. During image acquisition, all species were anesthetized with the volatile anesthetic isoflurane (2% in O_2_) (Fig. 1A) or MS-222 in the case of the sturgeon (Fig. 1B). The MS-222 was provided via oxygenated freshwater provided via tubing to the mouth. Before imaging, the eyes were dilated with 1% tropicamide and 2.5% phenylephrine (Akorn, Inc., Lake Forest, IL, USA). Because the animals used for in vivo OCT/OCTA imaging were not available for histology, the histology of retinas of these species reported in the literature were used for comparison with in vivo retina OCT images.

Representative ocular parameters of all imaged species, including eye's pupil size, eye maximum numerical aperture (NA_max_), eye focal length (f), total optical power in diopters, and axial eye length obtained from literature, are provided in Table 1. These parameters were used to design (choose) the sample arm imaging optics (tube and scan lens pair) for each species.

**Table 1.** Representative Ocular Parameters of the Imaged Species

Species	Pupil Size (mm)	NA_max_	Eye Focal Length (mm)	Total Optical Power (D)	Axial Eye Length (mm)
Mouse^17^^,^^18^	2	0.49	1.9	520	3.3
Rat^19^	3	0.43	3.3	300	6.1
Owl^20^	13.3	0.38	17.2	58	28.5
Opossum^21^	6	0.44	5.1	196	10
Fish^22^	2.1	0.44	1.9	532 (in air)	3.6

### SS-OCT System With a Flexible Imaging Head for In Vivo Retinal and Choroidal Imaging

For in vivo imaging, all animals were placed on a specially designed platform (OcuScience Inc., Henderson, NV, USA) (Fig. 1C). A custom-designed gimbal scanning head and holder of the OCT imaging system (with galvo-scanners mounted on linear X-Y-Z translational stages and imaging optics unit) allowed the flexible rotational and translational adjustment of the OCT imaging beam with respect to the animal eye (Figs. 1C, 1D).

A sample arm optics comprising of scan lens (focal length, f_s_) and tube lens (focal length, f_o_), delivers a collimated beam to the eye pupil, resulting in an axial focal spot that covers both retinal and choroidal layers. Table 2 lists the different focal lengths of the achromats chosen for imaging of each species. To allow detection of the retinal image for each of these configurations the reference arm length had to be adjusted accordingly to match distance to the retina.

**Table 2.** OCT Sample Arm Optics Chosen for Imaging of Different Animals

Species	f_s_ (mm)	f_o_ (mm)
Mouse	50	10
Rat	50	20
Owl	75	45
Opossum	50	20
Sturgeon	50	10

**Figure 2.**
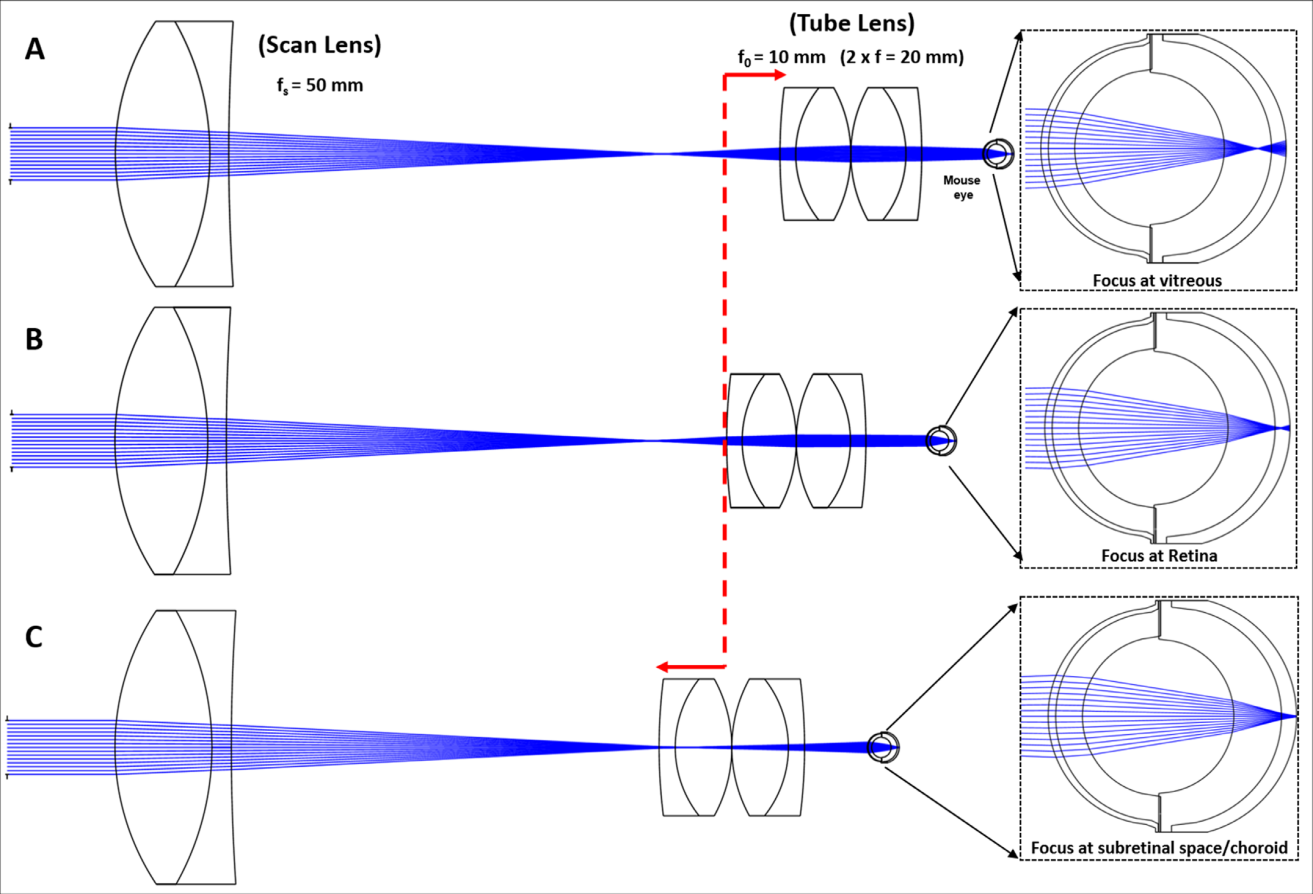
Zemax simulation of the retinal illumination on a mouse eye model, demonstrating the axial focal shift with respect to the axial position of the tube lens. (**A**) Focus at vitreous when tube lens is away from scan lens. (**B**) Focus in the retina when both lenses are separated at the sum of their focal distances (4f configuration). (**C**) Focus in the subretinal/choroidal region when tube lens is placed closer to scan lens.

The relative axial position of the tube lens in the telescope that optically conjugates X-Y galvo scanners with imaged eye pupil controls imaging beam collimation and thus can significantly alter the axial position of the focal plane in the eye. Figure 2 show a Zemax simulation of the retinal illumination of a mouse eye model, illustrating the apparent axial shift in the focus plane inside the eye with the change of axial position of the tube lens. It is evident from the Figure 2A that the focal spot shifts toward the vitreous when the tube lens is moved away from scan lens. When these two lenses are spaced at the sum of their nominal focal lengths (4f configuration), the eye is illuminated with a collimated beam, and in case of emmetropic eye the beam is focused on the retina (Fig. 2B). The focus is shifted to subretinal/choroidal region when the tube lens is moved toward the scan lens (Fig. 2C).

Thus the imaging beam collimation for imaging of different species could be easily adjusted, by shifting axial position of the tube lens with respect to the scan lens, during imaging session. The optimal position could be always found by observing, in real-time, changes in brightness/intensity in OCT cross-sectional images (B-scans) of retinal layers in each of the species. The scanning of OCT beam allows illumination of retina over varying field of view. Our simulations confirm that there is very little field dependent variation in the focal spot sizes for the beam sizes used in our experiments, and thus confirming that our imaging system is diffraction limited. However, when imaging animals, the distortions/shift in the focus at high eccentricities (edges of field of view) could be observed and are contributed by ocular aberration. Additionally, OCT scanning over large field of view can induce field curvature in some cases, where the optical pathlength across the scanning field doesn't match retinal curvature, resulting in curved retinal images. The large difference in the optical pathlength at the maximal scanning points cause the reduction in the OCT signal (sensitivity) and eventually degrade the quality of the images near the edges of the field of view. The curvature of the retinal OCT images presented here was corrected during post-processing of the OCT data (discussed in next section).

Our SS-OCT system with an incorporated CUDA-enabled graphic processor unit (GPU) used for in vivo retinal and choroidal imaging has been reported previously.^23^^,^^24^ In brief, a swept-laser source (Excelitas Technologies Corp., Waltham, MA, USA) with a center wavelength of ∼1060 nm and a tuning range of ∼100 nm was used in the SS-OCT system. The imaging system provided an optical axial resolution of ∼7.5 µm (in the air), and the measured system sensitivity was 101 dB (power at the sample: 800 µW). The swept-source has a sweeping rate of 100 kHz, resulting in an acquisition speed of 100k A-scans/s. The power of the sample light incident on the cornea was measured as 800 µW. A raster scanning was performed to acquire OCT data from 360 consecutive locations (y-mirror positions or B-scan locations) across the fundus with repeated three scans at every B-scan location (BM scans = 3). Every B-scan was comprised of 360 axial scans (x-mirror positions or A-scans). The scan angle was approximately 50° × 50° for imaging the retina of all species. The fiberoptic interferometer allowed the back-reflected light from the retinal layers to interfere with a reference light reflected from a static mirror to produce spectral fringes. These spectral fringe signals were Fourier transformed to obtain the depth-resolved reflectivity profile (A-scan). The time-encoded interference fringes generated for every A-scan were detected by a balanced photodetector synchronously with the laser sweeping. The balanced detected signals were digitized using a 12-bit data acquisition card (ATS9350, Alazar tech) at a rate of 500 MS/s. A uniform k-clock signal from the source was used to resample the spectral fringe signal linearly in k-space. The real-time data acquisition and image reconstruction processes for real-time display were performed in CUDA-enabled GPU (GeForce GTX 680 4GB, NVIDIA Corp., Santa Clara, CA, USA).^25^^,^^26^ This high-performance data acquisition platform allowed the acquisition of a retinal OCT volume (raw data) of size 360 × 1080 × 1024 pixels in four seconds (360 A-scans, 1080 B-scans [3 × 360], 1024 depth pixels).

### OCT Data Processing and Image Registration Process

The 3D OCT raw data acquired with the SSOCT system was processed using a custom MATLAB code to reconstruct and extract B-scan images. This postprocessing included DC subtraction, dispersion compensation, Hann windowing, FFT, logarithmic transformation, and grayscale conversion.^27^^,^^28^ The repeated B-scans (BM-scans) from a single position were averaged to obtain the final averaged B-scan at that position. Before averaging, the interframe motion between BM-scans was corrected using an FFT- based subpixel registration technique.^29^ The inter-frame (axial) motion between the averaged B-scans within the OCT volume was corrected using the cross-correlation approach. A good quality reference B-scan was manually selected from the central retinal position. Starting with this reference B-scan, the neighboring adjacent B-scans were registered using normalized cross-correlation in the axial direction. This axial correction was repeated until the entire OCT volume was corrected axially.

Following the registration process, 3D retinal OCT images were flattened based on an algorithm that operates on each cross-sectional image to segment its surface. Briefly, a gaussian and median filter was applied to the frames to reduce the speckle noise. Then, imaging thresholding was applied on the frames to remove the noise, especially above the surface portion of the image. Afterward, a Canny filter was applied that detected the edges in the image. Surfaces were then detected by finding the first non-zero elements in each A-line. Another median filter was applied to the array of edge locations from each B-scan to remove outliers in the detected edge and ensure a smooth surface profile. Once the surface positions were finalized on each cross-sectional frame, circular shifts on A-lines were performed in accordance with the distance between the location of the detected surface of that A-line and a pre-determined center depth.

### A 3D Rendering of OCT Data and Retina Thickness Calculation

An OCT en face fundus image showing blood vessels and the optic nerve head was generated by axially summing a 3D-OCT volume of size 360 × 360 × 1024 pixels. The volumetric rendering of the retina OCT images of all species was performed with the “volume viewer” function available in ImageJ software.

The average retinal thickness of each species was estimated across the volume using a custom MATLAB program based on a graph-cut segmentation approach. The 3D graph-cut algorithm based on the minimum-cut/max-flow algorithm was used to segment and inner limiting membrane (ILM)/NFL layer and retinal pigment epithelium (RPE)/Bruch's membrane (BrM) complex of the retina.^30^ The differences in the location of these layers on a micrometer scale provided the average and standard deviation of the retinal thickness of each species.

### In Vivo Extraction of the Retina and Choroidal Vasculature Using SS-OCTA Imaging

A speckle-variance OCT (SVOCT) analysis was performed to generate the OCTA images. SVOCT identified retinal microvasculature by calculating the interframe intensity variance (BM-scans = 3) using serially acquired structural OCT images. OCTA images were displayed with en face visualization (maximal intensity projection). The contrast of the OCT en face image was enhanced via background subtraction and contrast stretching using ImageJ software.

We used the following terms to indicate the retinal circulation, aligned to the current knowledge of retinal anatomy gained from histological studies^31^^,^^32^:

Superficial vascular plexus: characterized by a dense irregular meshwork of vessels composed of larger arteries, arterioles, capillaries, venules, and veins primarily in the NFL/ganglion cell layer.
Intermediate capillary plexus: comprises capillaries composed of vertical and oblique segments and located near the top and bottom of the inner plexiform layer (IPL).
Deep capillary plexus: comprises capillaries arranged in a one-dimensional laminar configuration and located between the inner nuclear layer (INL) and the outer plexiform layer (OPL).
Choroidal vasculature beds: The choroid is a vascular bed that supplies nutrients and oxygen to the RPE and outer layers of the retina. It consists of larger vessels and a highly fenestrated capillary bed known as the *choriocapillaris*.

## Results

The capabilities of the developed SS-OCT system for in vivo structural and angiography imaging of retinas across phyla allowing for longitudinal evaluation of terrestrial, aquatic, and aerial animals are demonstrated here. The volumetric, cross-sectional, and fundus projections of the retina OCT images, en face OCTA images of retinal and choroidal vascular plexuses, and quantitative assessment of the retinal thickness of different species are presented in the following subsections.

### In Vivo OCT and OCTA Images of a Long Evans Rat

**Figure 3.**
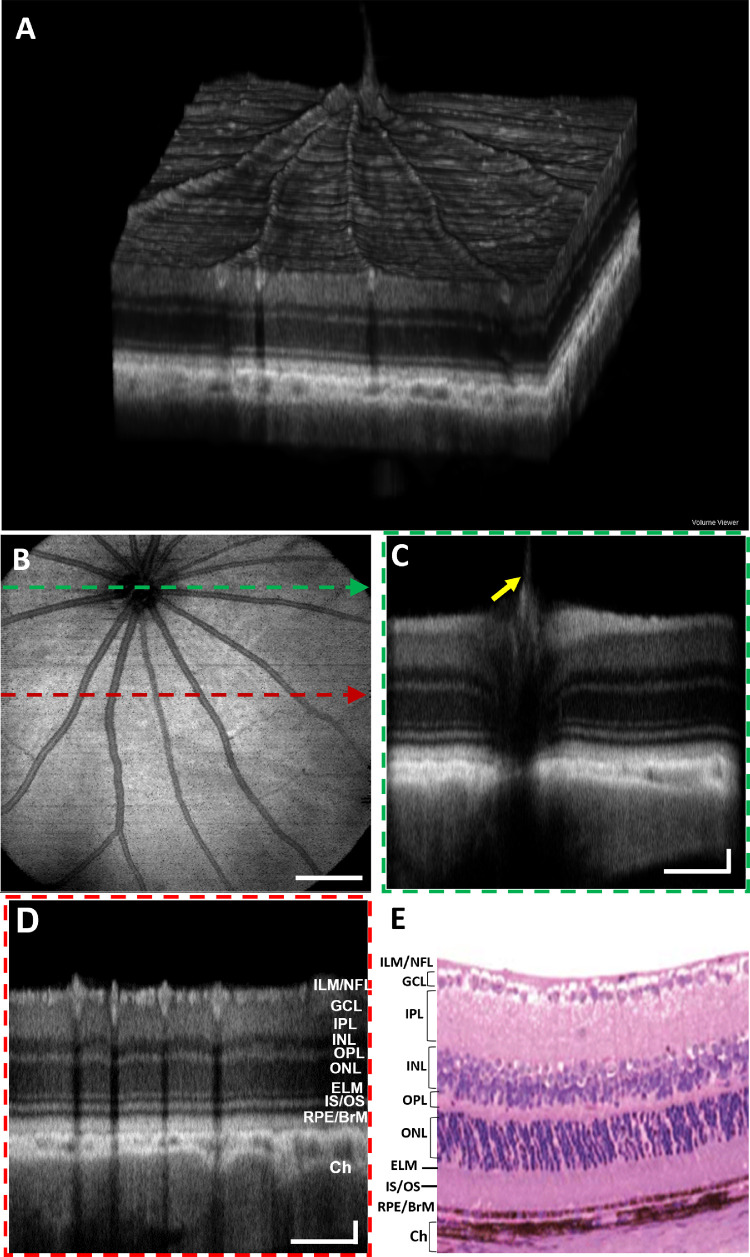
OCT images of a Long Evans rat. (**A**) Volumetric rendering of the 3D OCT data. (**B**) En face OCT fundus image. (**C**) B-scan extracted over the ONH as indicated by a *dashed green arrow* in (**B**). (**D**) B-scan extracted over the peripheral retina as indicated by a *dashed red arrow* in (**B**). (**E**) Representative histological section of a rat retina.^33^ *Scale bars*: Vertical = 400 µm; Horizontal = 50 µm.

Figure 3A shows a rendering of 3D OCT data from a Long-Evans rat retina. Figure 3B shows the OCT en face projection fundus image visualizing the blood vessels, NFL striations, and the optic nerve head (ONH). Figures 3C and 3D depict the B-scans acquired from the locations across the ONH (indicated by green dashed lines on Fig. 3B) and a distant location from ONH (red dashed lines on Fig. 3B), respectively. Figure 3E shows a representative histological structure of the age-matched rat retina reported by Chen et al.^33^ All primary retinal layers were visible in the OCT B-scans. The brightness (intensity) of each retinal layer depends on the scattering/reflecting properties of the corresponding tissue. The highly reflective top layer of the retina represents the ILM/NFL interface, which separates minimally scattering vitreous from the relatively highly backscattering retina. A very thin layer just below the NFL represents the ganglion cell layer (GCL) and exhibits relatively low backscattering of the GC somas. The next thick higher scattering layer can be distinguished as a bright band and corresponds to the IPL. A dark band below the IPL represents the INL with low backscattering of the somas of bipolar, horizontal and amacrine cells. Layers below INL are the highly backscattering OPL, with the low backscattering outer nuclear layer (ONL) comprising the photoreceptor somas, respectively. A thin reflective band distal to the ONL represents the external limiting membrane (ELM). A bright band posterior to the ELM, represents the photoreceptor inner segment (IS) and outer segment (OS) junction. The high reflectance at IS/OS junction is caused by the abrupt transition in the refractive index from the IS ellipsoid to the OS.^34^^,^^35^ A thick reflective band posterior to the photoreceptor IS/OS junction is the RPE/ BrM complex. This region represents the interdigitation of the photoreceptors' OS tips and the melanosomes containing RPE cell processes that extend into the OS layer.^36^ BrM, located at the bottom of this complex, separates the retina from the choroid. A distal region of high reflectivity corresponds to the choroid. Another important feature of the rat retina is the protruding hyaloid artery remnant (yellow arrow). The representative histological section of a Long Evans rat retina (Fig. 3E) is provided for comparison with *in vivo* OCT B-scans.

**Figure 4.**
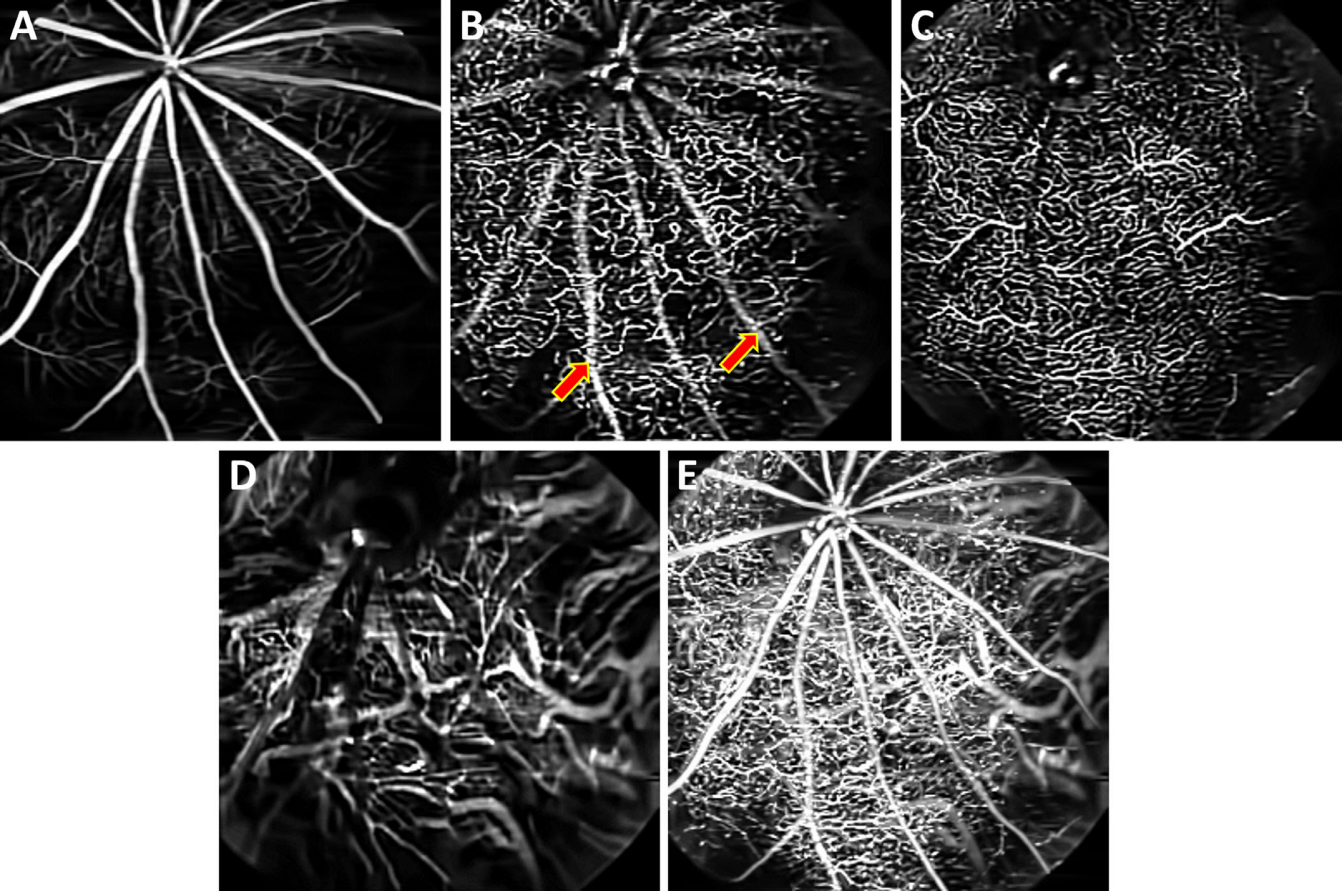
Depth-resolved OCTA en face images of a Long Evans rat. (**A**) Superficial vascular plexus at the NFL/GCL. (**B**) Intermediate capillary plexus at the IPL. (**C**) Deep capillary plexus at the OPL. (**D**) Choroidal vasculature. (**E**) OCTA en face projection (maximal intensity projection) image of retina and choroid vasculature. *Red arrows*: OCTA projection artifacts.

Figures 4A–4E show the en face images of the superficial vascular plexus at the NFL/GCL, intermediate capillary plexus at the IPL, and deep capillary plexus at the OPL, respectively. The choroidal blood vessels located below the RPE/BrM complex are shown in Figure 4D. Figure 4E shows the depth projected posterior vasculature of the retinal and choroidal vasculature.

OCTA projection artifacts, which are commonly observed during imaging, can be readily identified by examining sequential en face images at different depths (red arrows in Fig. 4). Projection artifacts will cause superficial vessels to appear in en face images, below the vessel, as seen in Figure 4B (red arrows). We have not applied any projection artifacts removal method to showcase the vascular images obtained by standard OCTA processing.

### In Vivo OCT and OCTA Images of a Mouse (C57BL/6J)

**Figure 5.**
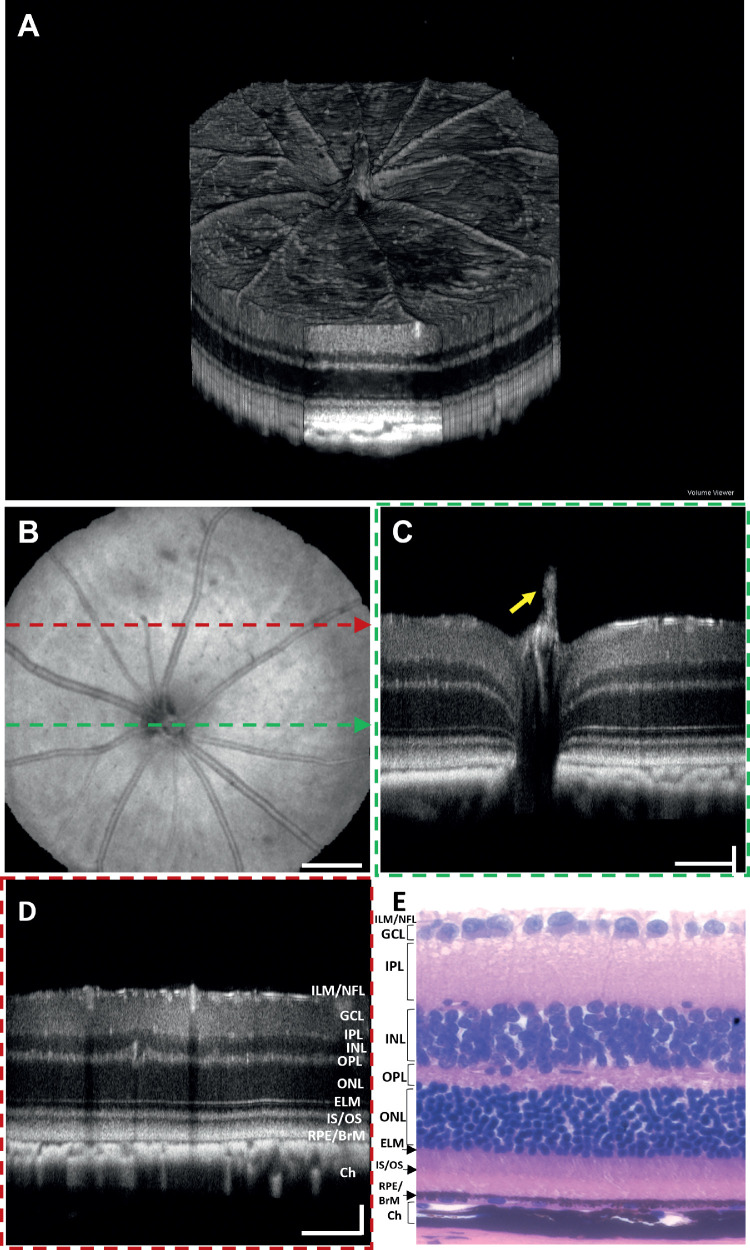
OCT and OCTA images of a mouse (C57BL/6J). (**A**) Volumetric rendering of the OCT data. (**B**) En face OCT fundus image. (**C**) B-scan acquired over the ONH as indicated by a *dashed green arrow* in (**B**). (**D**) B-scan acquired over the peripheral retina as indicated by a *dashed red arrow* in B. (**E**) Representative histological section of a C57BL/6J mouse retina.^37^ (*Scale bars*: Vertical = 300 µm; Horizontal = 50 µm).

Figure 5A represents the 3D visualization of the OCT image of a mouse retina. The OCT en face fundus image and the OCT B-scans of a mouse retina are shown in Figures 5B–D, respectively. All major retinal layers such as NFL/ ILM, GCL, IPL, INL, OPL, ELM, IS/OS, RPE/BrM complex, and choroid are evident from the B-scan images. Similar to the rat retina, a remnant of the hyaloid artery also appeared to extend into the vitreous body. Different retinal layers of a C57BL/6J mouse retina visualized in vivo using OCT are compared with a representative histological section (Fig. 5E), demonstrating good correlation between the retinal layers observed *in vivo* and in histology cross sections.^37^

**Figure 6.**
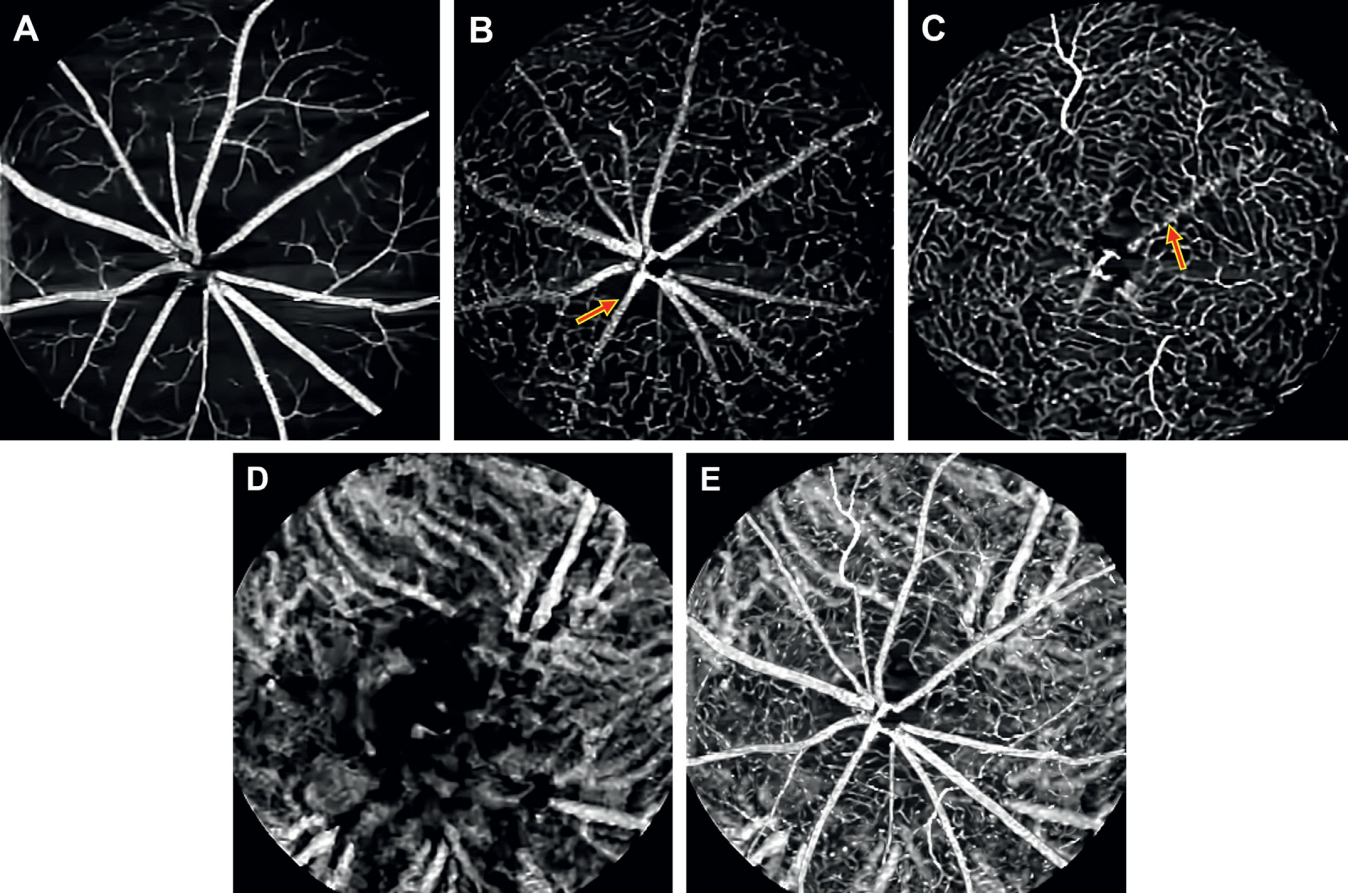
Depth-resolved OCTA en face images of a mouse (C57BL/6J) retina. (**A**) Superficial vascular plexus at the NFL/GCL. (**B**) Intermediate capillary plexus at the IPL. (**C**) Deep capillary plexus at the OPL. (**D**) Choroidal vasculature. (**E**) OCTA en face projection (maximal intensity projection) of retina and choroid vasculature. *Red arrows*: OCTA projection artifacts.

Figures 6 represent the en face images of the superficial vascular plexus at the NFL/GCL, intermediate capillary plexus at the IPL, and deep capillary plexus at the OPL, respectively. The thick choroidal blood vessels of the choroid are shown in Figure 6D. Figure 6E represents the en face projected OCTA images of the retinal and choroidal vasculature.

### In Vivo OCT and OCTA Images of a Gray Short-Tailed Opossum

**Figure 7.**
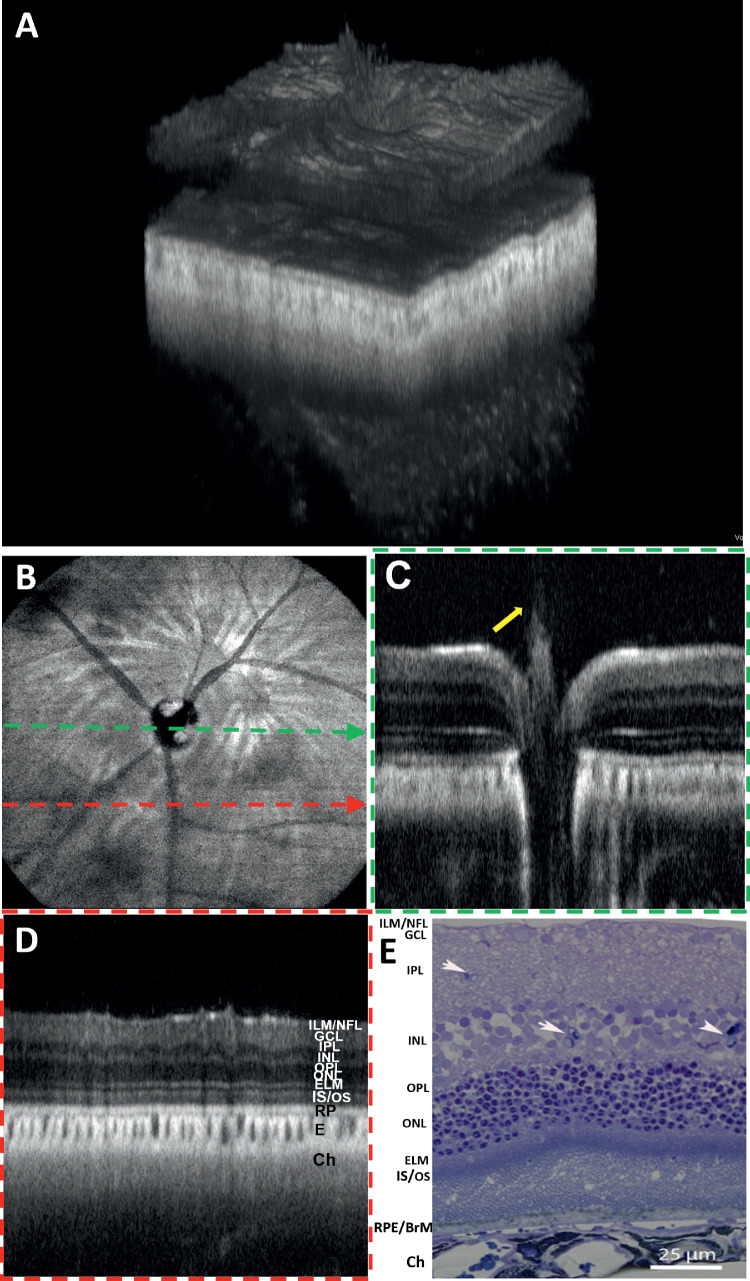
OCT and OCTA images of an opossum centered on the ONH. (**A**) Volumetric rendering of the OCT data. (**B**) En face OCT fundus image. (**C**) B-scan over the ONH as indicated by the *green dashed arrow line* in (**B**). *Yellow arrow*: hyaloid artery remnant. (**D**) B-scan acquired peripheral to the ONH as indicated by the *red dashed arrow* in (**B**). (**E**) Representative histological semithin resin section of a gray short-tailed opossum retina stained with toluidine blue.^38^

The 3D visualization of OCT volume of an opossum retina is shown in Figure 7A. The larger superficial retinal vessels produce prominent elevations on the retinal surface (Fig. 7A). The OCT B-scans acquired over the ONH (green dashed line in Fig. 7B), and a location distant from ONH (red dashed line in Fig. 7B) are shown in Figures 7C and 7D, respectively. The retinal layers such as the NFL/ILM, GCL, IPL, INL, OPL, ONL, ELM, IS/OS, RPE/BrM complex, and choroid are distinctively visible in the OCT B-scans. It is evident from the B-scans that the opossum has a unique choroidal morphology characterized by the thick choroidal vasculature passing parallel to the retina surface.

A representative histological section (Fig. 7E) of a gray short-tailed opossum retina, showed similar retinal laminations consistent with in vivo OCT B-scans.^38^ The histology image further demonstrates the paired retinal vessels (white arrows in 7E) in the opossum retina.

**Figure 8.**
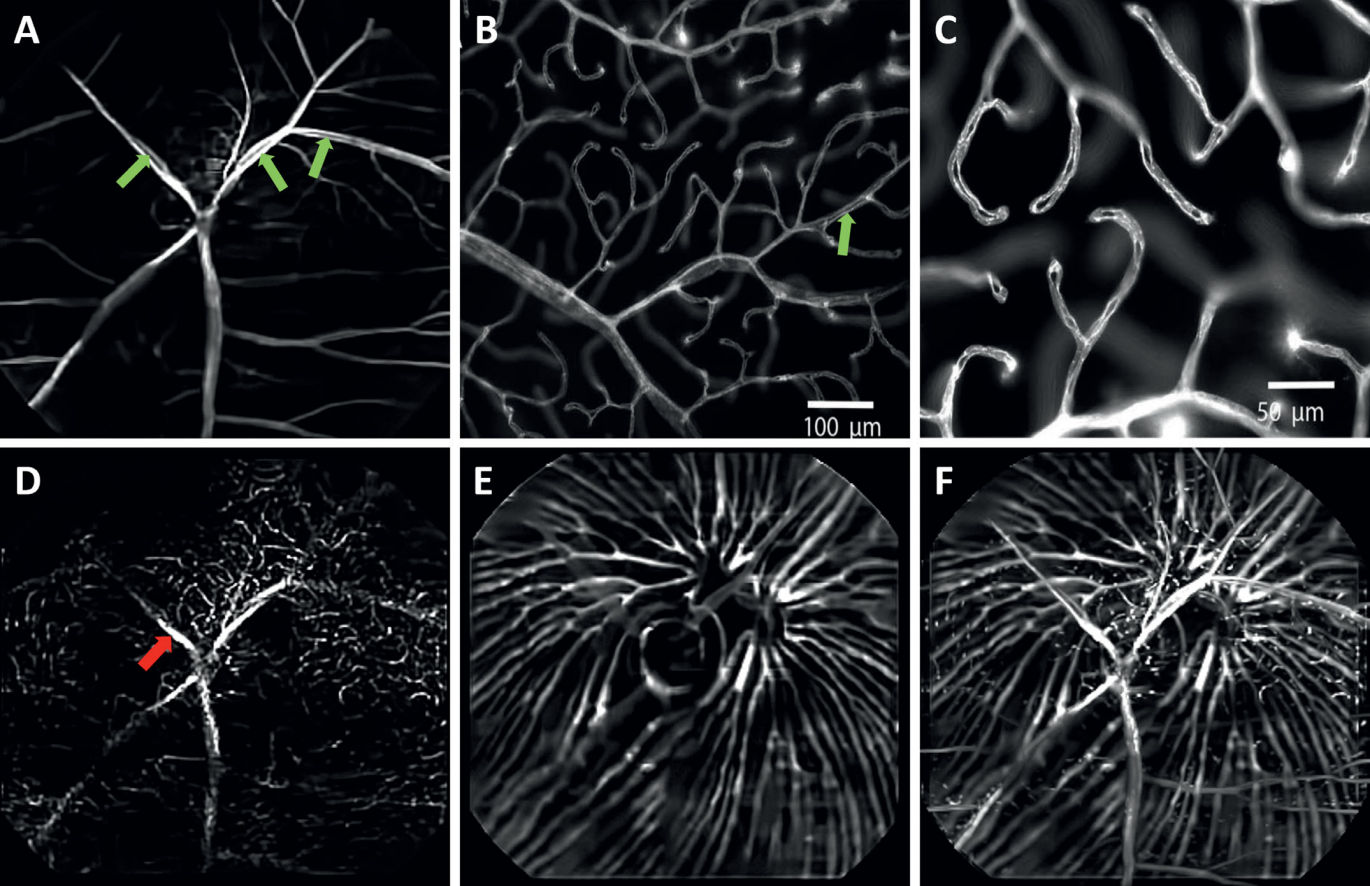
OCTA images of Opossum retina. Depth-resolved OCTA en face images: (**A**) Superficial vascular plexus at the NFL/GCL. (**B**, **C**) Representative fluorescent microscopy images of the superficial retinal vasculature from retinal wholemounts.^38^ (**D**) Deep capillary plexus at the OPL. (**E**) Choroidal vasculature. (**F**) Depth-projected (maximal projection) en face OCTA image of the retinal and choroidal vasculature. *Green arrows*: Paired blood vessels. *Red arrow*: OCTA projection artifact.

Figure 8 shows the en face images of the multiple vascular beds in the opossum retina. The in vivo OCTA images highlight the paired superficial retinal vessels in the opossum retina (green arrows in Fig. 8A). These large radially arranged pairs emerge from the optic disc and extend toward the periphery (Fig. 8A). A representative ex vivo confocal fluorescence microscopy of DiI perfused retinal wholemounts from a gray short-tailed opossum (Figs. 8B and 8C) shows the paired vasculature in superficial and their branching patterns more clearly.^38^ The termination of these paired capillaries ended in a blind end capillary loop (Fig. 8C). These larger superficial vessels traverse in the NFL and GCL and extend deeply before appearing to terminate at the level of the INL.

Figure 8D shows the OCTA en face image of the OPL. The OCTA en face image of the unique choroidal vessel architecture of a gray short-tailed opossum is shown in Figure 8E. The maximal intensity projected en face OCTA images of the whole retinal and choroidal vasculature is shown in Figure 8F.

### In Vivo OCT and OCTA Images of a Great Horned Owl

**Figure 9.**
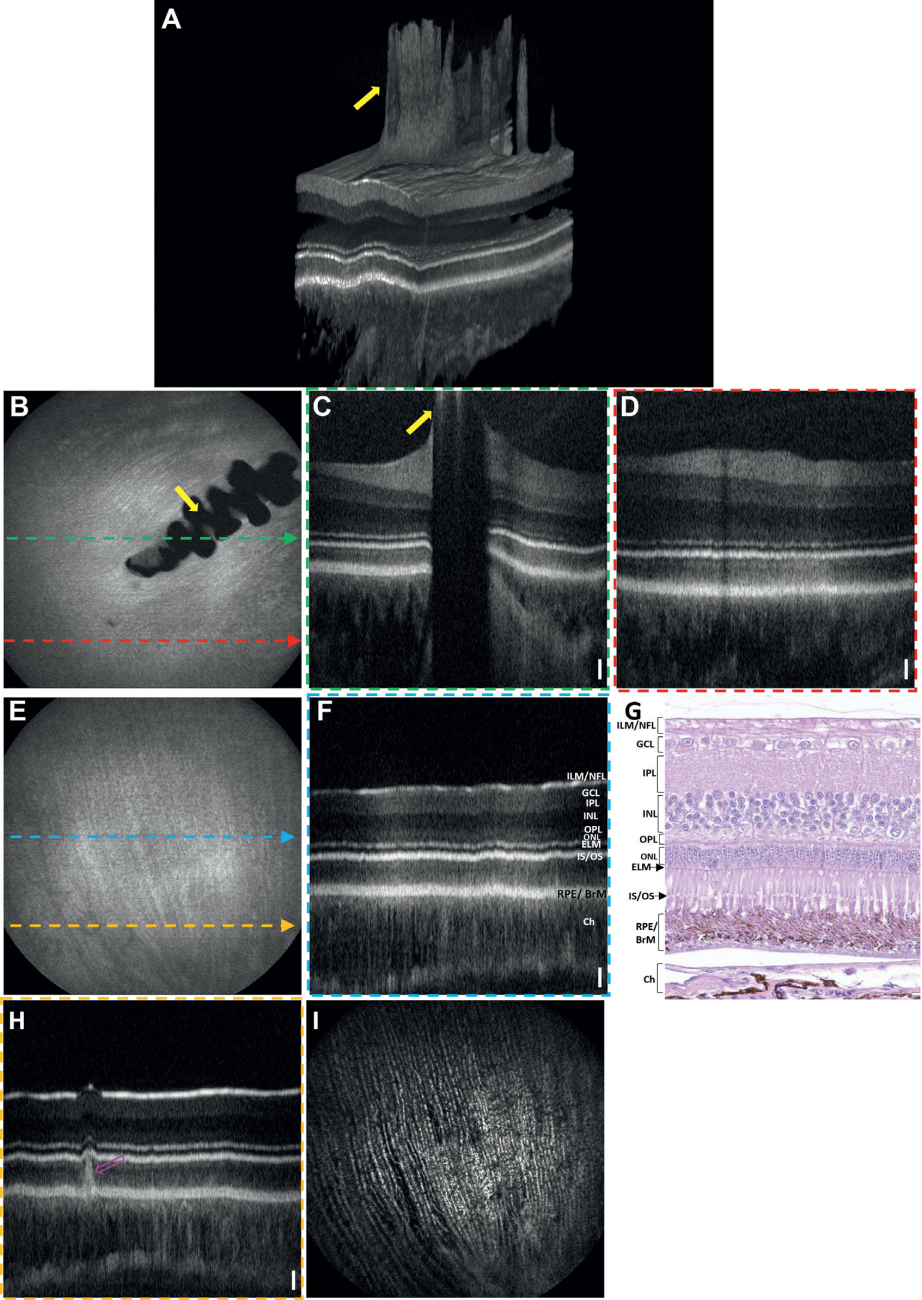
OCT and OCTA images of a great owl. (**A**) Volumetric rendering of the owl retina. (**B**) En face OCT intensity projection fundus image of the retina including the pecten (indicated by a *solid yellow arrow*). (**C**) B-scan extracted across the pecten as indicated by a *dashed green arrow* in (**B**). The *solid yellow arrow* shows the pecten protruding into the vitreous. (**D**) B-scan extracted at a position distant to the pecten as indicated by a *red dashed arrow* in (**B**). (**E**) En face OCT intensity projection fundus image of the peripheral retina. (**F**) B-scan corresponding to the location represented by the *blue dashed line* in (**E**). (**G**) Representative histological section of a great horned owl retina.^39^ (**H**) B-scan corresponding to the location represented by the *orange dashed arrow* in (**E**). OCTA en face image: (**I**) Depth-projected (average) OCTA image of the choroid. The *pink hollow arrow* indicates a subRPE lesion (reminisce a focal druse found in human retinas). *Scale bars*: Vertical = 50 µm.

A 3D volume rendering of the OCT images of a great horned owl retina is shown in Figure 9A. The en face OCT intensity projection fundus image of the owl retina and overlying pecten oculi are shown in Figure 9B. Figures 9C and 9D are the OCT B-scans acquired from the locations crossing the pecten (green dashed arrow in Fig. 9B) and distant to the pecten (green and red dashed arrow in Fig. 9B), respectively. Figure 9E represents an en face OCT fundus image of an owl retina corresponding to a peripheral retinal region. Figures 9F and 9G depict the OCT B-scan corresponding to the location of the blue dashed line and a histology cross-section image of a great horned owl retina reported in the literature. 9H depicts the B-scans corresponding to the location indicated by the orange dashed arrow lines in Figure 9E. All OCT B-scans visualized different retinal layers: NFL/ILM, GCL, IPL, INL, OPL, ONL, ELM, IS/OS, RPE/BrM complex, and choroid. The NFL/ILM layers are found to be highly scattering in images 9F and 9H. The lack of similar high reflectance of NFL/ILM in 9C and 9D might be caused by the oblique incidence of OCT beam. The OPL appears to be weakly scattering, whereas the ELM and IS/OS junctions and RPE/BrM complex appeared as the bright bands in all B-scans. A lesion found in the subRPE space seen in Figure 9H (pink hollow arrow) is reminiscent of a druse observed in human retinas. A representative histology cross-section of a great horned owl retina is shown in Figure 9G.^39^ The retinal layers in the histology cross-section are closely correlated with the OCT B-scan images. OCTA images confirm that an avascular retina is overlying a highly vascularized choroid, shown as an en face image in Figure 9I.

### In Vivo OCT and OCTA Images of a White Sturgeon

**Figure 10.**
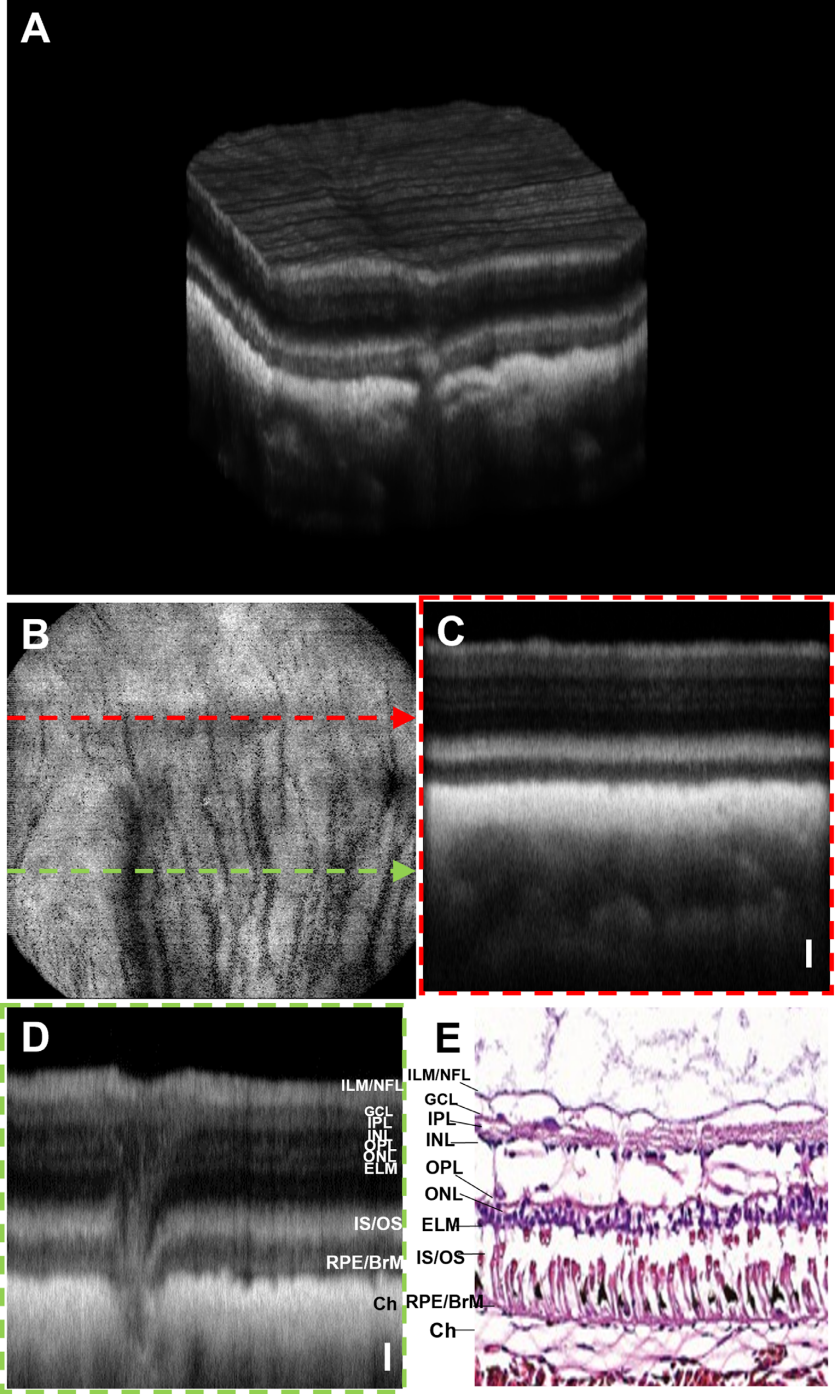
OCT and OCTA images of a white sturgeon. (**A**) Volumetric rendering of the OCT data. (**B**) En face OCT fundus image. (**C**) B-scan acquired across the ONH as indicated by a *green dashed arrow* in (**B**). (**D**) B-scan acquired across the peripheral retina indicated by a *red dashed arrow* in (**B**). (**E**) Representative histological section of a white sturgeon retina.^40^ *Scale bars*: Vertical = 50 µm.

A 3D visualization of the OCT volume and OCT fundus image of a sturgeon retina are shown in Figures 10A and 10B, respectively. The B-scans from the retinal location depicted by the arrow distant to the ONH (red dashed arrow in Figure 10B) and crossing ONH (green arrow in Figure 10B) are provided in Figures 10C and 10D, respectively. The retinal morphology of the sturgeon includes different retinal layers ILM/NFL, GCL, IPL, INL, OPL, ONL, ELM, and IS/OS and RPE/BrM complex, which are evident from OCT B-scans. Interestingly, an elongated ONH (parallel to the retina surface) was found in the retina of the sturgeon. Figure 10E depicts a representative histological section of a white sturgeon retina showing similar overall retinal architecture to the other presented species, which is consistent with the in vivo OCT B-scans.^40^

**Figure 11.**
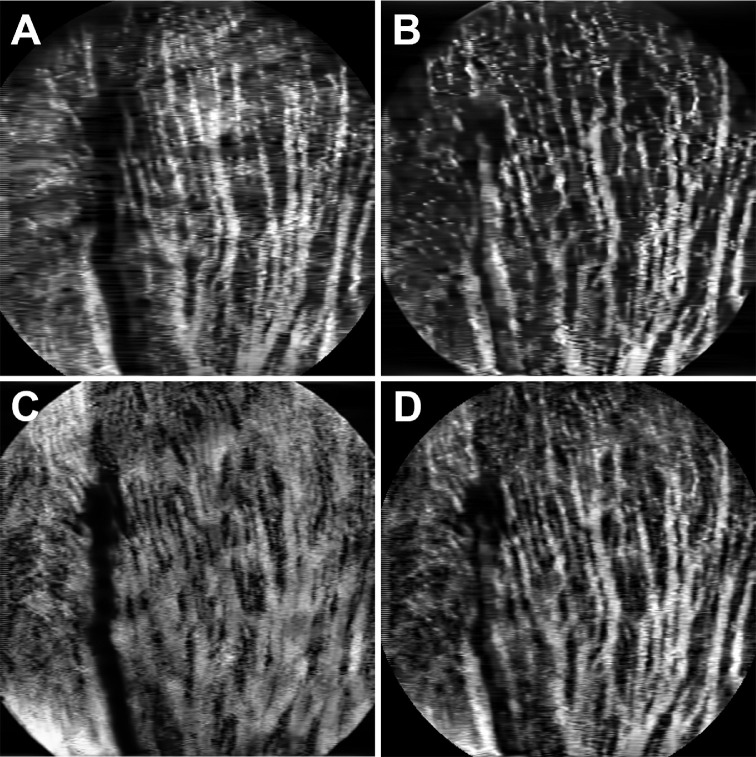
Depth-resolved OCTA images of white sturgeon. (**A**) Superficial vascular plexus at the NFL/GCL. (**B**) Deep capillary plexus at the OPL. (**C**) Choroidal vasculature. (**D**) En face depth-projected (maximal intensity projection) OCTA image of all vascular beds.

En face OCTA images of the superficial vascular plexus, and deep capillary plexus of the retina are shown in Figures 11A and 11B, respectively. The en face image of the choroidal vasculature is shown in Figure 11C. Figure 11D shows an en face depth projection of the OCTA signal from all vascular beds.

### Assessment of the Retina Thickness

**Figure 12.**
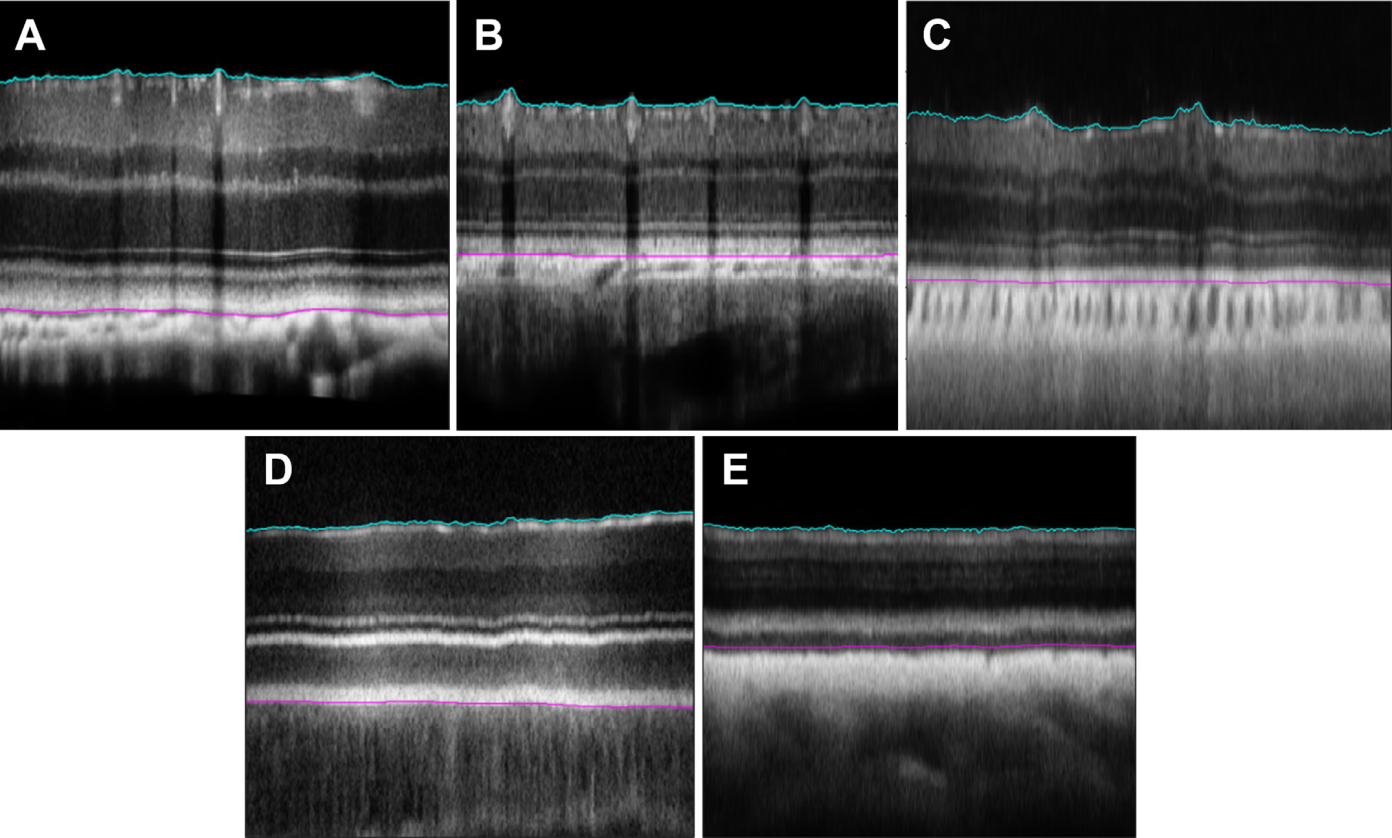
Representative retinal OCT B-scans showing segmentation of the NFL and RPE layers in (**A**) mouse (**B**) rat (**C**) opossum (**D**) owl, and (**E**) sturgeon. The *pink line* represents the RPE/BrM complex. The *cyan line* represents the ILM/NFL interface.

Representative OCT B-scan images showing retinal layer segmentation (ILM/NFL and RPE/BrM complex) used for calculating the mean and standard deviation of the retinal thickness of each species are shown in Figure 12. A custom-developed 3D graph-cut-based retinal segmentation and thickness calculation program allowed for an accurate calculation of the average retinal thickness of each species from their acquired OCT volume data (See Table 3).

**Table 3.** The Average Retinal Thickness Measured for the Imaged Species

Species	Retina Thickness (µm), Mean ± SD
Mouse	210 ± 10
Rat	186 ± 15
Owl	260 ± 24
Opossum	138.5 ± 14
Sturgeon	142 ± 12

## Discussion

Anatomic study of the eye has historically relied almost exclusively on in vitro evaluation (e.g., histology). Although this affords a great many benefits, it is limited by the examination of postmortem tissue, need for highly skilled preparation, and incompatibility with longitudinal study. Evaluation of delicate structures poses a greater challenge, and, even with exceptional care, artifacts are commonly observed. These artifacts may be misinterpreted, lead to errant conclusions of disease-dependent changes, and fail to appropriately represent in vivo physiology.^41^ With the advent of OCT and OCTA, imaging of the retina and choroid in vivo is now possible, which has the potential to provide a more detailed and accurate evaluation of the retinal anatomy. Most of the standard OCT imaging devices available in the market are built for human ocular imaging applications. A modified version of these systems, typically used for pre-clinical research, suffer by a lack of flexibility for imaging a wide range of animals. Most commercial OCT instruments available in markets for clinical and pre-clinical research are based on SD-OCT configurations with an a-scan acquisition speed below 100k A-scans per second (Table 4). The in-house built retinal OCT system presented here, based on SS-OCT technology, possesses several inherent advantages over the SD-OCT system. The incorporation of a custom-designed gimbal scanning head and holder for the OCT imaging optics in our system enabled flexible imaging of animal eyes including those 1/10 the size of a human eye simply by changing the imaging optics (scan and tube lens pair) and adjustment of reference arm position. In typical retinal imaging of animals, accurate focusing of OCT beam over retina/choroid regions was often challenged by the selection of scan and tubing lenses, their relative positions, and the unique ocular features (parameters) of each species. In our system, any shift in focal plane could be easily corrected via adjusting tube lens axial position (enabled by flexible tube mounting) and simultaneous real-time assessment of retinal images.

**Table 4.** Comparison of Commercial OCT Systems and SS-OCT System for Animal Imaging

OCT Systems	Configuration	Center Wavelength (nm)	Axial Resolution (µm in Air)	Speed (A-Scans/Sec)	FOV
SD-OIS; Bioptigen	SD-OCT	840	4	17k	50°
Envisu OCT; Leica Microsystems	SD-OCT	870	3	32k	70° for human 50° in animal
Spectralis SD-OCT	SD-OCT	870	7	40k	15°
3D-OCT 2000; Topcon	SD-OCT	840	6	50k	45°
CIRRUS HD-OCT; Model 5000 (Zeiss)	SD-OCT	840	6.75	68k	36°
iVivo VET-OCT/Occuscience	SD-OCT	<840	6	80k	60°
iVue; Optovue	SD-OCT	840	5	80k	21°
OCT system presented in this article	SS-OCT	1060	7.5	100k	50°

A comparison of the specifications provided in Table 4 shows that our SS-OCT system yields the highest speed of retinal imaging (compared to available SD-OCT systems) over a relatively large field of view. Imaging retina with a longer center wavelength 1060 nm relative to 840 nm in commercial systems, allows for a better tissue penetration and reduced effects of water dispersion (vitreous and aqueous humor), enabling imaging of outer retinal structures across species.^42^^,^^43^ The lower axial resolution of our SS-OCT system relative to the other standard systems is a direct consequence of the limited bandwidth of the light source used. By using a swept source with wider sweeping range, an improved axial resolution could be achieved. Similarly, the field of view of our SS-OCT system could be further enhanced by redesigning of the scanning head and use of an appropriate galvo scanner and adjustment of the electronics and software settings. Although our prototype SS-OCT system makes use of a source with limited sweep rate of 100 kHz, retinal OCT imaging at much faster rate (in Megahertz range) would be possible with high-speed swept sources available in the market as well as those demonstrated by different research groups.^10^^,^^12^ Such video rate volumetric OCT imaging of the retina with multi-MHz A-scan rates will allow for the averaging of multiple OCT volumes to significantly enhance the structural and OCTA image quality.^44^^,^^45^

With the current, relatively simple configuration, our SS-OCT system readily images the retina and choroid across vertebrate phyla, including the first published OCT and OCTA images of the opossum, great horned owl, and sturgeon. All major retinal layers, including NFL, GCL, IPL, INL, OPL, ONL, ELM, IS/OS, RPE/BrM complex, and choroidal region of all the species, were distinctly visible in their OCT cross-sectional images. The OS tips of the photoreceptors, however, were not resolved in OCT images, which is due to the limited axial resolution of the system and the strong scattering from RPE melanosomes obscuring the interdigitated OS tips. It is noteworthy that the in vivo structural morphology assessed with the SS-OCT system was closely correlated with previously reported histological sections, although direct comparison of layer thickness was not feasible because of potential artifacts occurring during the preparation of histological sections.^41^^,^^46^

The en face visualization of OCTA images provided valuable information regarding the architecture of retinal and choroidal vasculature in all species. Across the imaged species, there was general preservation of retinal architecture with significant similarities in vasculature noted among more closely related vertebrates. In most of the species, the projection artifacts inherent to OCTA could be seen while examining en face images at different depths. These artifacts cause superficial vessels to be seen in en face images, which are below the vessel, although the vessel is not located in that layer. These artifacts hinder the accurate quantitative assessment of the various vessel parameters. The projection artifacts can be significantly suppressed via subtracting them from images below, albeit this can introduce artifacts of its own.^47^^,^^48^

As has been published previously, the retinal OCT and OCTA images of rats and mice (both members of order Rodentia) demonstrate a similar structure morphology and vasculature of capillary anastomotic network.^49^^,^^50^ One commonality observed between the rat, mouse, and opossum (a marsupial) was the presence of a hyaloid artery remnant. The hyaloid artery is a developmental vessel extending from the optic nerve to the anterior segment, which regresses during normal development. Although histologic evaluation would be necessary to confirm this observation, this highlights the potential value of longitudinal imaging in individual animals both in ocular development and pathology (e.g., persistent fetal vasculature).^51^

Unlike the capillary anastomotic networks visible in rat and mouse retinas, which is the most common pattern observed in mammalian retinal vasculature, the superficial vessels in several marsupials (including the gray short-tailed opossum) display a distinct morphology. Retinal arteries and veins originate from the nerve as pairs that join distally to form hairpin end loops (Figs. 8A–C). This vascular pattern, although also present in the central nervous system in these species, is not found elsewhere in the body nor in most mammals.^52^^,^^53^ One prior study has shown this via fluorescein angiography, but was unable to resolve the vascular plexuses or to image the choroid.^38^ This paired vessel architecture with visualization of the superficial and deep plexuses in addition to choroidal vasculature is visible here in vivo for the first time (Figs. 8A–E). Notably, the choroid shows the more common capillary anastomotic structure similar to the mammals reported here. This difference in architecture may result from the massive metabolic demands placed on the choroid, which may not be adequately met by a paired vessel system.

Despite maintaining the same arrangement of retinal layers as found in other vertebrates, birds have developed a markedly different approach to meeting the oxygen requirements of the inner retina.^54^ As can be seen in the OCTA of the great horned owl reported here (Figs. 9B, 9E), the avian retina is avascular. This is consistent with the reported avascularity in birds including raptors.^55^^–^^57^ The avascular retina limits the scattering of light as it travels from the retinal surface to the photoreceptors and may contribute to many members of this class having excellent acuity.^58^ The pecten oculi is a highly vascularized body found in the avian eye that is attached to the linear optic nerve head and protrudes far into the vitreous body. It delivers nutrients to the vitreous and avascular avian retina.^59^ This vascular structure is larger and more elaborated in diurnal birds than in nocturnal ones and can be seen extending into the vitreous body of the great horned owl presented here.^56^^,^^59^ Interestingly, the great horned owl has a choroidal vascular structure (Fig. 9I) similar to the other species presented here, likely indicating a similar means of meeting the vascular needs of the outer retina.

All retinal layers in the white sturgeon were observed on OCT and correlated with the histology. The quality of retina OCT B-scans from sturgeon was compromised due to the deteriorated axial and lateral resolutions and sensitivity (Figs. 10C, 10D). This is caused by the strong optical dispersion and aberration induced by the portion of water in which the fisheye was immersed during imaging. An elongated ONH was observed in OCT fundus images, albeit histological validation was not available. SS-OCTA could further reveal the thick, dense vascular plexus of the sturgeon retina and choroid, demonstrating the blood supply to both the inner and outer retina. The capillaries in the inner retina were not visible in the OCTA images, potentially because of the deterioration of spatial resolution caused by the optical dispersion and aberration.

The SSOCT imaging further allowed the in vivo assessment of the retinal thickness of all species. Although one animal per species was imaged, the average retinal thickness varied across the species, indicating the biometric difference in their retinal anatomy. Improved accuracy of retina thickness (average and SD) would be possible from the measurements from multiple animals per species.

We have demonstrated a custom-built SS-OCT imaging system with incorporated flexible imaging arm for in vivo retinal OCT and OCTA imaging of small terrestrial, aquatic, and aerial vertebrates animals. The capability of the developed SSOCT imaging probe for structural (retina morphology), blood flow (OCTA) and quantitative (retina thickness) imaging of multiple species was established. The SS-OCT/SS-OCTA imaging revealed in vivo visualization of the unique structural morphology of the retina, retinal vascular plexus, and choroidal vascular plexus across vertebrate species. Although this pilot study makes use of a single animal from each species, a more detailed quantitative analysis with a greater number of animals in addition to comparative retinal histology from the same retina (used for in vivo imaging) may provide additional insight. Additionally, SS-OCT imaging allowed for the longitudinal investigation including in vivo retinal thickness measurements. These quantitative assessments can serve as a normative reference for future studies and illustrate a standardized method of assessing in vivo retinal structure in common animal models of eye disease. A more comprehensive investigation of retinal functions would be possible with in vivo retina OCT imaging of these animals at a near-cellular-level in conjunction with electrophysiological and behavioral approaches. In summary, SS-OCT/SS-OCTA is a powerful imaging tool in vision research that facilitates the investigation of different morphological features of the retina and choroid across species in addition to increasing the ease by which less-commonly investigated animals may serve as models to further our understanding of the human disease.
